# Towards routine 3D characterization of intact mesoscale samples by multi-scale and multimodal scanning X-ray tomography

**DOI:** 10.1038/s41598-022-21368-0

**Published:** 2022-10-08

**Authors:** Ruiqiao Guo, Andrea Somogyi, Dominique Bazin, Elise Bouderlique, Emmanuel Letavernier, Catherine Curie, Marie-Pierre Isaure, Kadda Medjoubi

**Affiliations:** 1 Synchrotron SOLEIL, 91190 Saint-Aubin, France; 2 Université Paris-Saclay, 91190 Gif-sur-Yvette, France; 3 Institut de Chimie Physique, Université Paris-Saclay, CNRS, 91405 Orsay, France; 4 UMR S 1155, Sorbonne Université, 75020 Paris, France; 5 Institut National de la Santé et de la Recherche Médicale (INSERM), Sorbonne Université, 75013 Paris, France; 6 Physiology Unit, Hôpital Tenon, AP-HP, 75020 Paris, France; 7 IPSiM, Univ Montpellier, CNRS, INRAE, Institut Agro, 34000 Montpellier, France; 8 IPREM, Université de Pau Et Pays de l’Adour, E2S UPPA, CNRS, 64053 Pau, France

**Keywords:** Imaging techniques, Scanning probe microscopy

## Abstract

Non-invasive multi-scale and multimodal 3D characterization of heterogeneous or hierarchically structured intact mesoscale samples is of paramount importance in tackling challenging scientific problems. Scanning hard X-ray tomography techniques providing simultaneous complementary 3D information are ideally suited to such studies. However, the implementation of a robust on-site workflow remains the bottleneck for the widespread application of these powerful multimodal tomography methods. In this paper, we describe the development and implementation of such a robust, holistic workflow, including semi-automatic data reconstruction. Due to its flexibility, our approach is especially well suited for on-the-fly tuning of the experiments to study features of interest progressively at different length scales. To demonstrate the performance of the method, we studied, across multiple length scales, the elemental abundances and morphology of two complex biological systems, Arabidopsis plant seeds and mouse renal papilla samples. The proposed approach opens the way towards routine multimodal 3D characterization of intact samples by providing relevant information from pertinent sample regions in a wide range of scientific fields such as biology, geology, and material sciences.

## Introduction

Complex scientific problems in biology, earth-, environmental, and material sciences are inherently multi-scale. This requires the investigation of nanoscale features and functionalities within system-representative mesoscale samples to link those to emergent properties and functionalities at larger scales. This triggers an ever-increasing demand for new analytical tools capable of providing spatially resolved multi-scale information on intact, highly heterogeneous, or hierarchically structured samples in situ or *in operando*. Scanning hard X-ray imaging and tomography techniques are ideally suited to tackle this challenge due to their large penetration depth and inherently multimodal nature, where complementary information on the elemental distribution, morphology, crystalline structure, and chemical speciation can be obtained simultaneously. Moreover, these non-invasive scanning techniques provide straightforward access to multiple-length scale experiments. Recent developments in fast continuous scanning, data acquisition^1–4^, and the high flux obtained at modern synchrotron-based hard X-ray nanoprobes have opened routine access to scanning 2D multimodal imaging. Amongst the scanning techniques, the high analytical sensitivity of X-ray Fluorescence (XRF) imaging provides unique possibilities in several scientific fields to study the role and fate of trace elements^5–13^. However, the unambiguous interpretation of 2D elemental distribution maps is not always straightforward or feasible and is especially problematic in the case of thick, complex samples. As such, the scientific community is highly demanding the extension of XRF imaging and other complementary scanning techniques to 3D tomography. Scanning XRF and multimodal tomography, where measuring projection images at different projection angles permits the reconstruction of the internal features by adapted reconstruction methods, provides unambiguous information about the internal elemental distributions in the context of sample morphology, crystalline structure, chemical speciation, etc.^2,14–21^. Up to now, the lengthy acquisition time necessary for these experiments^2^ at 3rd generation synchrotrons has been one of the practical difficulties to its comprehensive utilization. Indeed, even if emerging sparse tomography techniques permit boosting the measurement throughput^15,22,23^ by compromising spatial resolution, overcoming the time constraint remains a challenge at 3rd generation synchrotrons.

Meanwhile, at the dawn of 4th generation synchrotron sources, routine 3D scanning X-ray tomography is becoming within reach. Indeed, the two orders of magnitude larger flux available at 4th generation hard X-ray nanoprobes boosts the speed of scanning tomography measurements proportionally, paving the way towards high-throughput scanning X-ray tomography of mesoscale samples^24^. Hence, the implementation of robust, user-friendly scanning tomography workflow is crucial for the routine application of these techniques, similarly as has been published recently for high-throughput electron tomography^25^. Such workflow imposes flexible and fast on-site data processing and tomographic reconstruction adapted to the different number of projections (sparse and high-resolution tomography), diverse data quality (e.g., high and low-level elemental abundances, missing wedge), different imaging modalities (e.g., XRF, X-ray absorption, X-ray diffraction), and the possibility of adapting the field of view and spatial resolution to the examined phenomenon by using multi-scale or local tomography. Some recent developments tackle part of these requirements by sample-type specific processing of multimodal tomography data sets^15,18^, also in a semi-automatic way in the case of similar processing requirements^17,26^. A sparse sampling approach followed by sophisticated data processing has also been reported^15,27^. However, according to our knowledge, a robust, holistic approach addressing all requirements of flexible multi-scale and multimodal scanning 2D/3D X-ray tomography does not exist yet.

In the present paper, we introduce such a robust workflow for scanning multi-length scale XRF-tomography and complementary modalities. The presented workflow has been developed and implemented at the Nanoscopium beamline^21^ of SOLEIL Synchrotron and includes semi-automatic data reconstruction. The proposed reconstruction algorithm yields good reconstruction data quality for diverse scientific fields with no need for parameter readjustment depending on the sample type. As the first step of this approach, sparse tomography provides a 3D overview of the entire meso- or microscale sample. The reconstructed sparse tomograms, containing relevant information to the investigated scientific problem, are used to choose pertinent regions for high spatial resolution single slice tomography, projection imaging, and local tomography. This approach permits optimizing the scanning tomography experiments and obtaining relevant information from pertinent sample regions in 2D or 3D during a user project. Thanks to recent developments^3,22,28,29^, this method paves the way towards statistically significant 3D studies, similar to those already available in full-field X-ray tomography^30^, and electron tomography^25^.

The performance of the workflow is demonstrated through the study of two highly heterogeneous mesoscale samples. A 700 μm thick wild-type *Arabidopsis thaliana* seed, widely used as a model organism for plant biology studies, has been measured by sparse XRF tomography followed by high-resolution 2D tomography of some virtual slices and 2D projection imaging. The multi-length scale study of mesoscale renal papilla samples is crucial to investigating pathological calcification^31^. This study highly profited from high-resolution local XRF tomography of a micron-sized calcification sphere chosen from the reconstructed sparse tomograms.

## Results

### Multi-length scale scanning X-ray imaging/tomography workflow

**Figure 1:**
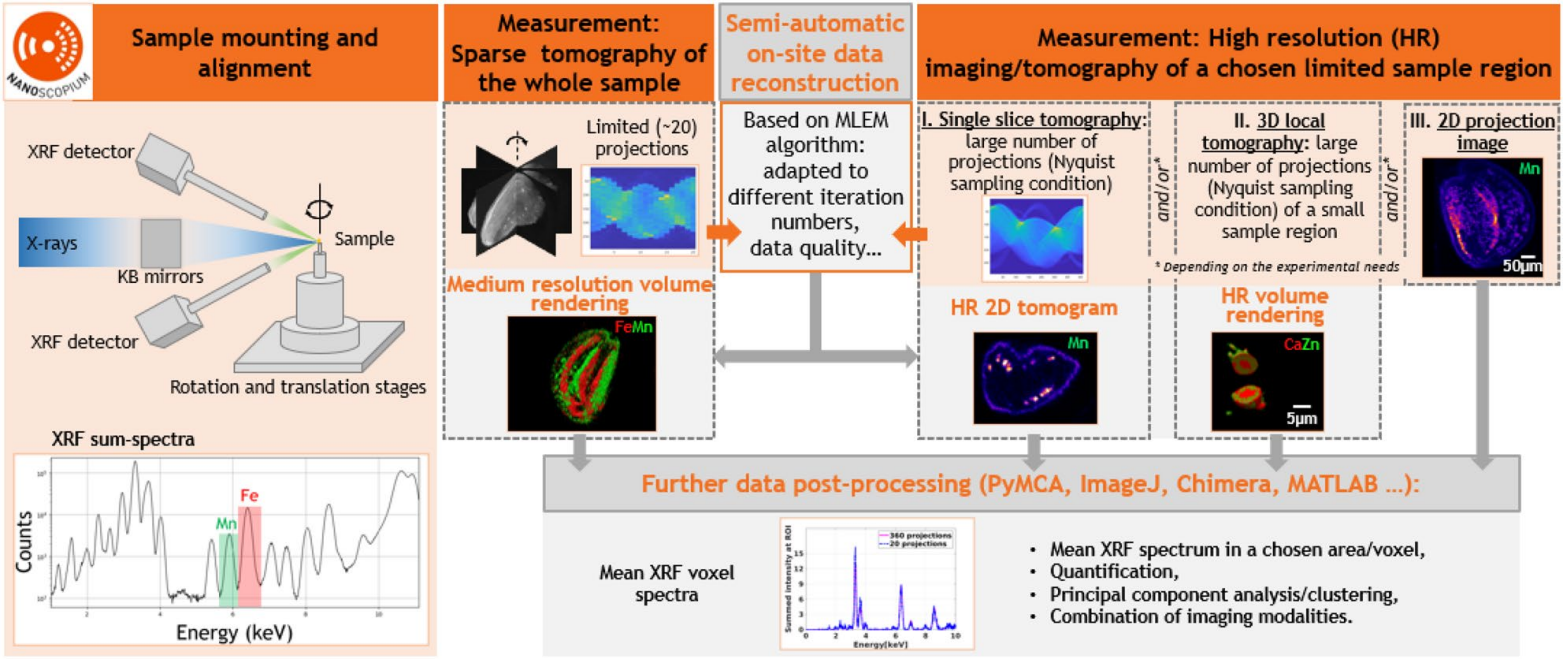
Semi-automatic multi-scale XRF and multimodal scanning imaging and tomography workflow implemented at Nanoscopium. After sample mounting and alignment, sparse scanning tomography is performed on the whole sample. Some minutes after the experiment, medium resolution volume rendering is available for data interpretation by the proposed semi-automatic on-site data reconstruction. This permits optimizing the strategy of successive high-resolution (HR) measurements. The on-site reconstruction algorithm provides HR tomograms for data interpretation and further data processing some minutes after the HR experiments. The measurement and data reconstruction steps are included in orange and grey rectangles, respectively.

The workflow implemented at Nanoscopium for multi-length scale and multimodal scanning X-ray imaging and tomography experiments is presented in Fig. 1: the sample mounting and alignment are followed by sparse tomography of the whole sample, and then the visualization of the volume rendering of the reconstructed tomograms. The strategy of high-resolution (HR) measurements is based on these medium-resolution results. This workflow permits even users who are new to X-ray imaging and tomography, to accomplish all measurement and data reconstruction steps during their experiment. At Nanoscopium all user projects apply for XRF multi-scale imaging or tomography, which is often complemented by other modalities (absorption- or phase-contrast imaging/tomography, XANES, or XRD) to best tackle the actual scientific question. As such, in this paper, we have chosen scanning XRF tomography to demonstrate our approach.

As a first step, a sparse XRF/multimodal tomography measurement with 20 projection angles (see details in “Data acquisition” section) is followed by semi-automatic on-site tomography reconstruction. Direct visualization of a specimen’s reconstructed medium-resolution 3D elemental distribution and morphology enables immediate identification of information pertinent to the research project. This permits users to choose the strategy for succeeding in high-resolution (HR) measurements for studying the smallest sample features crucial to tackling the scientific problem^32^. This HR measurement can be 2D projection imaging, 2D single slice tomography, local scanning 3D tomography, or any combination of these. Notably, a reasonable trade-off must be made during a tomography experiment regarding acquisition time, spatial resolution, and the number of samples wished to be investigated. After the high-resolution experiment, an estimate of the spatial resolution achievable by different projection numbers can be obtained by the Fourier ring correlation (FRC) calculation (detailed in the “Methods” section). If the resolution of the first sparse tomography results does not meet the user's requirements, then a second sparse tomography measurement can be performed. This will start with an angular offset equal to the half angular step of the first sparse tomography experiment. The second optional sparse tomography, having the same projection numbers as the first, results in doubling the total number of projections and hence improving the spatial resolution. As demonstrated in Fig. 1, all these modalities can be reconstructed semi-automatically on-site during the experiment using predefined and pre-parameterized reconstruction algorithms with no need for interaction from the users. Hence, users can guide their experiments and redefine the scientific objectives on-the-fly depending on the results obtained on-site.

Moreover, the HDF5 data format of the reconstructed tomograms is fully compatible with widely used cross-platform freewares and open-source data analysis tools, such as PyMCA (XRF data processing), ImageJ, Chimera (imaging/tomography data processing), etc. As such, further on-site or post-experiment processing, such as extraction of mean XRF spectra, quantification, combined treatment of different imaging modalities, and multivariate statistical analysis (Principal Component Analysis, Cluster Analysis, etc.)^33^, can be obtained straightforwardly in any 2D/3D feature identified from the projection images or the reconstructed 2D/3D tomograms.

### Proof of principle test measurements: Wild-type *Arabidopsis thaliana* seeds

*Arabidopsis thaliana* is a weed native to Eurasia and Africa with a short life cycle (≥ 6 weeks). This annual plant is a popular model organism in plant biology due to the knowledge of its genome and the availability of numerous mutants. The wild type (Col-0) is commonly used in plant biological laboratory experiments and even in Space experiments to study genetics, evolution, and development of flowering plants. As a model plant, *Arabidopsis thaliana* is also a powerful tool to investigate metal homeostasis and nutrient distribution, relevant questions about the world’s food production and the agriculture industry. In this context, essential elements Fe and Mn in the plant seed are crucial for plant germination. Mn, involved in the photosystem II in chloroplasts, is needed for the vigour of germinating plants, and Fe is involved in various metabolic processes (respiration, photosynthesis…). These two elements have different physiological functions and different transporter pathways. Thus, differences are expected in their distribution and concentration within the seed. 2D XRF imaging is a powerful tool for locating trace elements in plants. However, assigning the measured metal distribution unambiguously to the grain’s ultrastructure can be challenging due to the several hundreds of microns information depth of Mn and Fe. Moreover, sample sectioning, required to study the internal elemental distribution by 2D scanning XRF imaging, can be intricate for small and hard samples such as *A. thaliana* seeds and can induce tissue alteration and artifacts.

XRF and multimodal tomography are useful complementary tools for determining metal concentrations and distribution in intact seeds with minimal sample preparation^16,34,35^.

### Sparse scanning X-ray tomography of a whole seed

**Figure 2:**
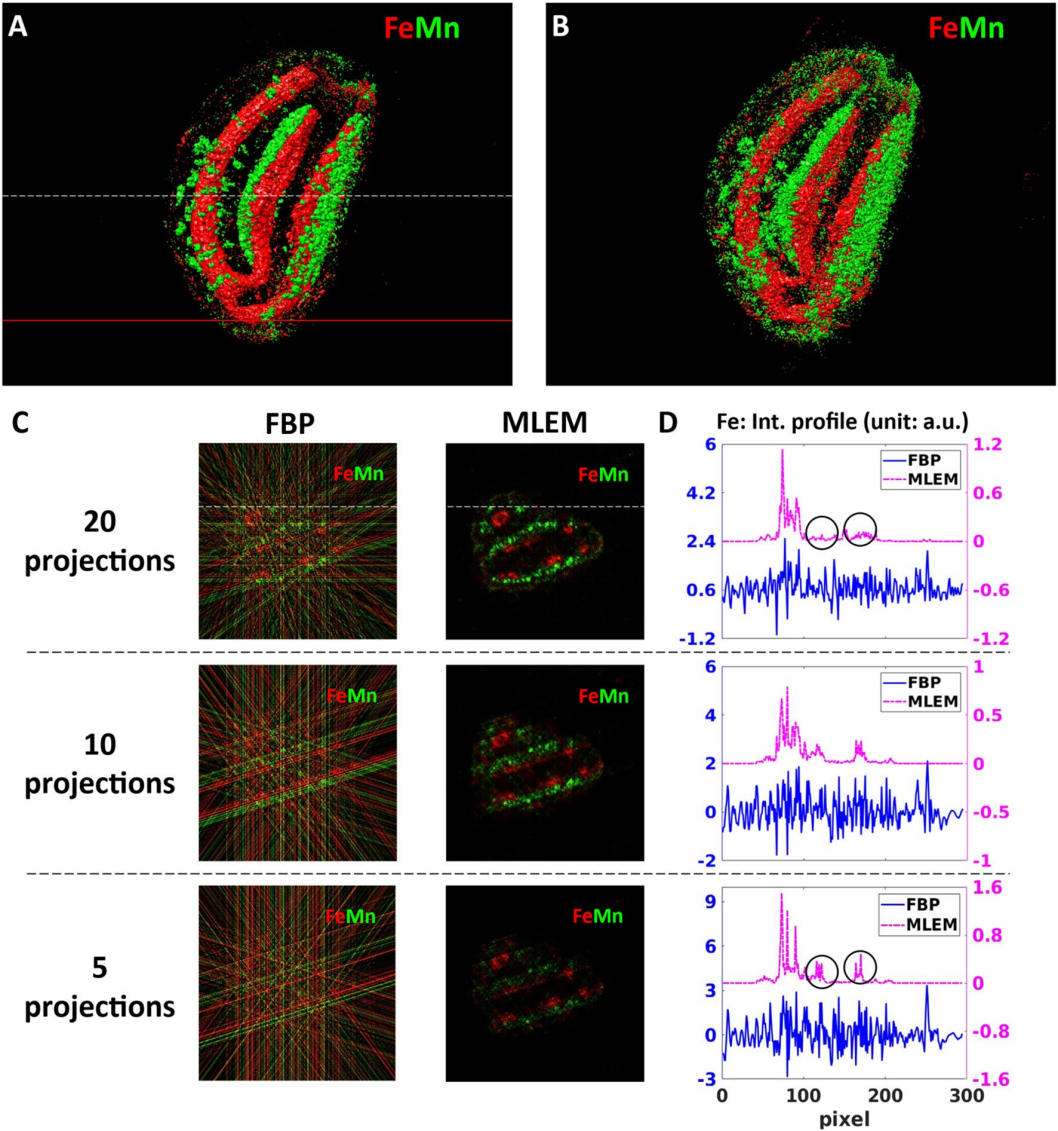
Reconstructed sparse 3D Fe and Mn tomograms and a virtual slice showing the internal Fe and Mn distributions. (**A**,**B**) volume rendering of the Fe and Mn tomograms reconstructed by MLEM from 20 (**A**) and 5 angular projections (**B**). The white dashed line indicates the altitude of the virtual slice shown in (**C**). The red line marks the altitude of the high-resolution single slice tomography shown in Fig. 3. (**C**) Comparison of the reconstructed results obtained by the FBP and MLEM algorithms in the function of the measured number of angular projections. The columns correspond to two different algorithms: filtered back-projection (FBP) and maximum likelihood expectation maximization (MLEM). The lines correspond to 20, 10, and 5 angular projections, respectively. (**D**) Comparison of the intensity profiles of Fe obtained by FBP and MLEM. The Fe intensity profiles were extracted along the white straight line indicated in (**C**).

We tested the performance of our tomography approach on wild-type *Arabidopsis thaliana* seeds. One advantage of using seeds, in general, is their low water content, resulting in lower amounts of radiation damage than more hydrated tissues. In order to obtain an overview of the elemental distribution of a whole mesoscale seed of 700 µm dimensions, we performed simultaneous sparse XRF (see Fig. 2) and absorption (Fig. S1) micro-tomography. The figure of merit of measuring a limited number of projections is to identify, within acceptable measurement times, the features of interest, in our case the distribution of Mn and Fe within different seed compartments. Since the total measurement time of a tomography experiment scales proportionally with the number of projections, it can be drastically reduced by measuring only a few angular projections. This also reduces the eventual radiation damage of the sample, which might be important in the case of scanning imaging of larger samples. However, the reduced number of projections should be chosen without significantly compromising the quality of the obtained 3D mesoscale tomograms. The reconstructed sparse 3D tomograms of Mn and Fe can be seen in Fig. 2A, B. It comprises 223 virtual slices and is calculated from 20 measured angular projections by the maximum-likelihood expectation–maximization (MLEM) algorithm (described in “Methods” section). It is clear from the reconstructed sparse tomograms that Fe is preferentially located in the provascular systems of the seed, and that Mn is mainly distributed in the abaxial area of both cotyledons, at the subepidermal level, as well as in the cortex area of the hypocotyl. These observations are in agreement with the results published in the literature^34,35^.

To optimize the measurement conditions and the corresponding reconstruction parameters of sparse tomography, we compared two different reconstruction algorithms, Filtered back- projection (FBP) and MLEM, in the function of the number of measured angular projections (5, 10, and 20). The comparison is demonstrated in Fig. 2C, D using the virtual slice marked by a white dashed line in Fig. 2A.

FBP algorithm is the standard solution for 3D tomography reconstruction due to its fast reconstruction time and easy implementation. However, because of the intrinsic nature of the algorithm, FBP has severe limitations in the case of noisy datasets and highly under-determined measurement conditions with a small number of projections^36^. This can be clearly seen in the first column in Fig. 2C, where the signal-to-noise ratio of all sparse tomograms obtained from 5, 10, and 20 projections is very low. Indeed, the strong streak artifacts of FBP, caused by the small number of projections, shadow the elemental distributions: the Mn and Fe distributions are hardly visible even in the case of 20 angular projections. In effect, as shown by the Fe intensity profiles (shown in Fig. 2D by the blue curves), these strong artifacts, resulting also in negative intensity values, are hiding the information on the Fe variation within the sample. As such, since FBP cannot significantly reduce streak artifacts at low number of projections, it is not adapted to sparse tomography reconstruction.

MLEM algorithm (second column of Fig. 2C) is superior to FBP in handling noisy datasets. Moreover, it includes the non-negativity constraint assumption. In our workflow, we included a simple automatic stopping criterion for the MLEM algorithm with the smallest possible noise as a figure of merit (as detailed in “Methods” section). This choice results in terminating the reconstruction process at small number of iterations (thus with short reconstruction time) introducing only a few, weak artifacts in the reconstructed tomograms. The reconstructed tomograms in Fig. 2C illustrate the robustness of this algorithm in treating limited number of projections. Moreover, with increasing number of projections the reconstruction artifacts of MLEM are becoming weaker. This is illustrated by the purple Fe profiles in Fig. 2D, where the strongest reconstruction artifacts marked by the black circles in the 5-projection tomogram (last line in D) diminished in the 20-projection tomogram (first line of D).

### High-resolution single slice tomography

**Figure 3:**
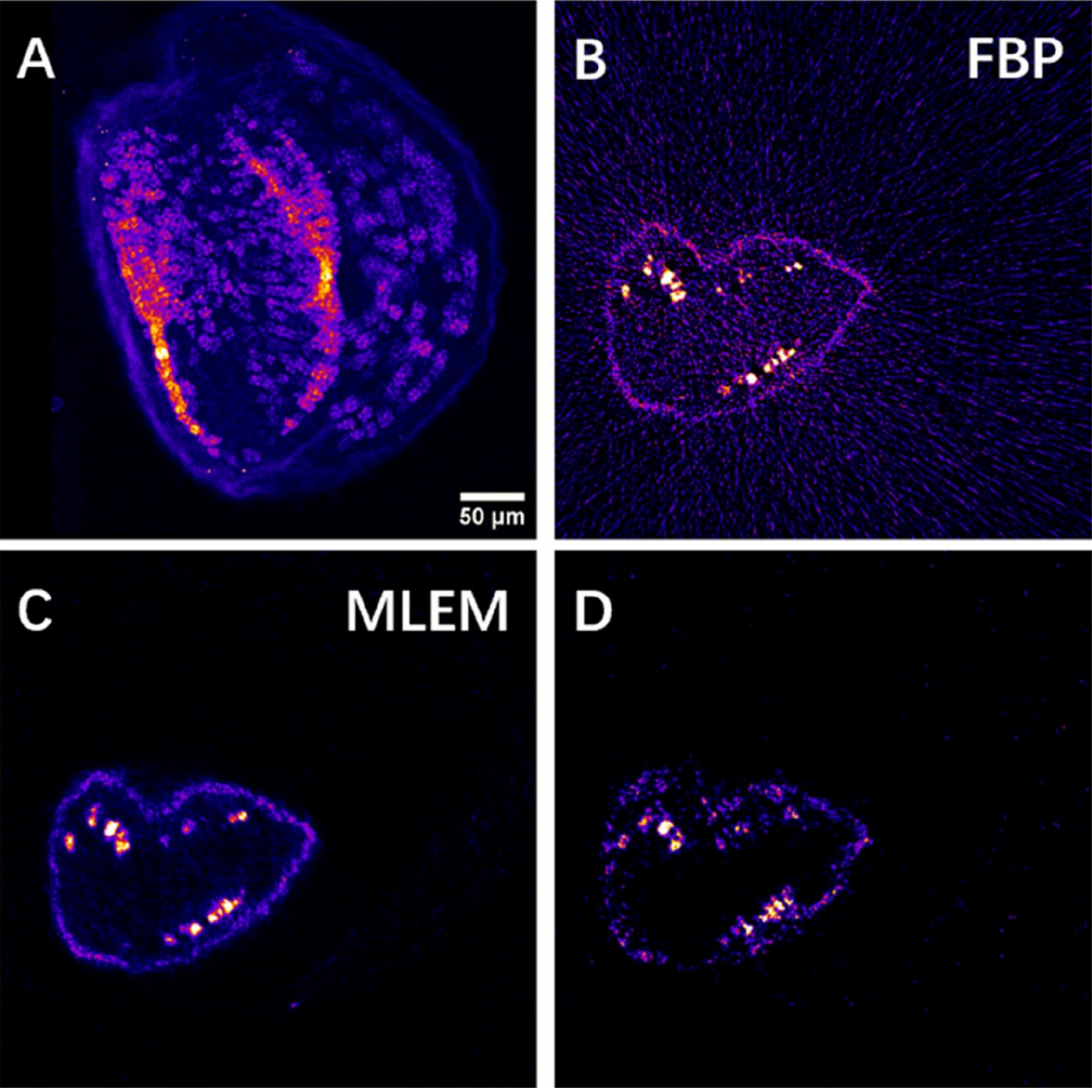
High-resolution 2D scanning XRF imaging and tomography of the Arabidopsis seed. (**A**) High-resolution 2D projection image of the Mn distribution at an appropriate angle chosen from the 3D sparse tomograms. (**B**,**C**) Reconstructed HR single slice of the Mn tomogram obtained by the FBP algorithm (**B**) and by the MLEM algorithm (**C**) measured at the altitude marked by the red line in Fig. 2A. (**D**) Mn sparse tomography reconstruction at the same slice obtained by MLEM.

The above-described 3D sparse tomography permits choosing the best angular position for high-resolution 2D projection imaging, as shown in Fig. 3A for Mn distribution. It also allows choosing the altitude(s) for high-resolution single slice tomography (Fig. 3B, C). This provides insight into the internal Mn and Fe distributions of subcellular features within intact seeds. We performed HR single slice XRF tomography at the altitude shown by the red line in Fig. 2A. The FBP and MLEM reconstructions of the internal 2D Mn distribution can be seen in Fig. 3B, C. We included for comparison the sparse tomogram of Mn obtained by MLEM in the very same virtual slice (Fig. 3D). The reconstructed features are in good agreement between the sparse (Fig. 3D) and high-resolution tomography (Fig. 3B, C) results. However, as expected, high-resolution tomography reveals fine details with improved spatial resolution, which are non-detectable or hardly identifiable by sparse tomography. For such HR tomography, the use of FBP (Fig. 3B) is straightforward and is the fastest reconstruction algorithm, where the computation time is proportional to the registered number of projections. However, even with 360 angular positions, there are non-negligible streak artifacts due to insufficient angular projections compared to the number of scanned pixels in this mesoscale sample (see Nyquist sampling condition in Eq. 3). The reconstruction result of the MLEM algorithm (Fig. 3C) provides better contrast. Moreover, the computation time of < 2 s/sinogram is significantly faster than the measurement time of some hours (and will be comparable with the measurement time of some minutes/sparse tomography at a 4th generation synchrotron). As such, the MLEM algorithm proved to be the best compromise for semi-automatic image reconstruction for both 3D sparse tomography and 2D high-resolution single slice tomography experiments.

### Spatial resolution of the tomograms reconstructed by MLEM

To estimate the spatial resolution of the tomograms reconstructed by the MLEM algorithm, we extracted two subsets with uniform angular sampling from the 2D high-resolution dataset. Taking the 2D high-resolution sinogram of 360 projections as an example, the full projection dataset was divided into two subsets, both having the same projection numbers. These two subsets were reconstructed independently by MLEM with the automatically determined iteration numbers. The two independent tomograms were then used for FRC analysis.

**Figure 4:**
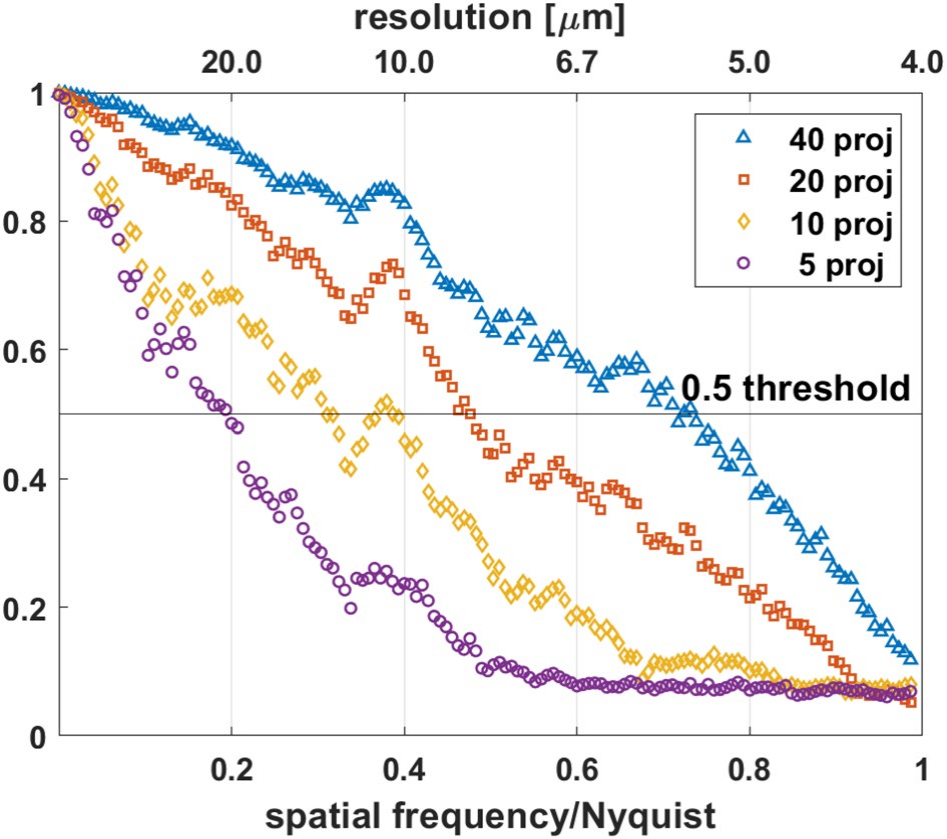
Spatial resolution estimation by Fourier ring correlation (FRC) method. Estimation of spatial resolution for tomograms reconstructed by MLEM from different numbers of projections chosen from the measured 360 angular projection dataset. The fixed 0.5 threshold was used for FRC analysis. The Nyquist frequency is 0.25 μm^−1^.

Figure 4 shows the FRC curves between the two tomograms reconstructed using a different number of projections from the same 2D high-resolution sinogram. The spatial resolution was determined at the intersection of the FRC curve and the fixed 0.5 threshold^37^. Table 1 shows the spatial resolution determined by the 0.5 threshold criterion in the function of the number of projections. The spatial resolution improves with the number of projections as expected (see Figs. S1, C and E). A resolution estimate of 8.4 μm was obtained for the 20-projection tomogram. The MLEM iterative method has resulted in a significant resolution improvement compared to the spatial resolution given by the Nyquist sampling condition.

**Table 1:** Estimation of the spatial resolution of sparse tomograms (reconstructed by the MLEM algorithm) by FRC analysis in the function of the measured number of projections.

Number of projections	FRC analysis: Resolution (μm)	Nyquist sampling condition: resolution (μm)
5	20.5	184.7
10	12.9	92.4
20	8.4	46.2
40	5.6	23.1

The spatial resolution defined by the Nyquist sampling condition is included in the 3rd column for comparison.

### Renal papilla sample

In order to test the performance of our workflow in missing wedge measurement conditions and for HR 3D local tomography, we investigated mouse renal papilla samples. Nowadays, the formation of renal stones affects 10% of the global population^38^. Most of the stones develop on Randall’s Plaque, a mineral deposit at the tip of renal papillae^39^. The major components of Randall’s Plaque are calcium phosphate apatite and amorphous carbonated calcium phosphate^40^. Therefore, the distribution of Ca indicates the position of calcification in the renal papilla sample. Studying the correlation and colocalization between Ca and other major and trace components permits to reveal those trace elements, that are involved in early-stage calcification processes. Understanding their role in the pathological process opens the way toward efficient prevention and treatment of the renal stone formation. The sample we used in the present study is a mouse papilla affected by calcium phosphate deposits similar to the human Randall's plaque (see^41^ for more details on the sample preparation).

### Sparse and high-resolution XRF tomography of renal papilla

**Figure 5:**
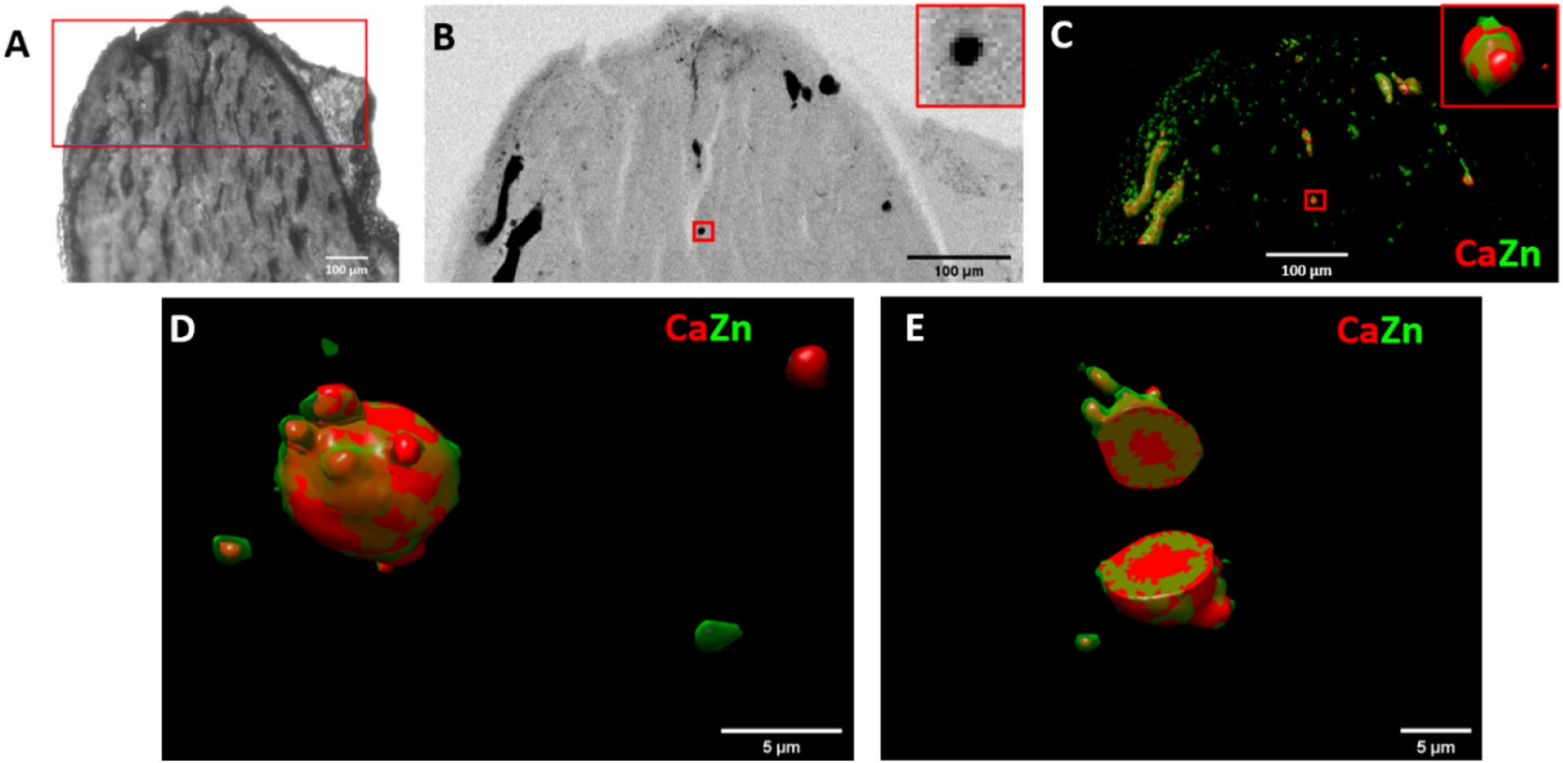
Sparse and local scanning X-ray tomography of a renal papilla sample. (**A**) Optical microscope image of the mouse papilla sample, mimicking human kidney calcifications. The scanned region is marked in red. (**B**) Transmission image of the tip of the sample. The small red rectangle in the middle marks the sample region chosen for local tomography. The zoom-in image of this region is inserted into the upper-right corner. (**C**) Sparse tomography reconstruction of Zn and Ca. The small red rectangle in the middle marks the sample region chosen for local tomography. The zoom-in image of this region is inserted into the upper-right corner. (**D**) Reconstructed 3D local tomogram of Ca and Zn of the calcification micro-sphere marked by the red rectangles in (**B**,**C**). (**E**) Cut-off view at the middle of the calcification micro-sphere presented in (**D**).

The semi-automatic workflow has been used to study the elemental abundancies within 50 µm thick slices of renal papilla samples (Fig. 5A, B). In this case, the fixation of the thin biological sample of ~ 500 µm lateral dimensions on a Si_3_N_4_ membrane caused a missing wedge of 2 × 28° in the tomography measurement. This poses a specific challenge to tomographic reconstruction^42^. This was successfully tackled by the MLEM algorithm, which proved to be the best compromise also for missing wedge tomography. 3D sparse tomography performed on the top of the sample showed substantial colocalizations between Zn and Ca (Fig. 5C) within dense sample regions revealed by simultaneous transmission imaging (Fig. 5B). However, the ~ 8.5 µm medium spatial resolution of sparse tomography does not permit to reveal the details of micrometer-sized Ca- and Zn-rich features, that are related to early-stage calcification process. Gaining insight, with high spatial resolution, into the elemental distribution of these micrometer-sized Ca and Zn containing spherical structures is crucial. We performed 3D local tomography around a chosen micro-sphere (red rectangles in Fig. 5B, C). The angular projection images were measured with 500 nm pixel size. Figure 5D, E show the volume rendering of Zn and Ca within the measured micrometer-scale calcification sphere. In the cut-off view of the 3D volume rendering in Fig. 5E the internal distribution of Zn and Ca is shown within the ~ 10 µm dimension calcification sphere. These results reveal that Zn is enriched within a few micrometers thick rim on the surface of the calcification micro-sphere. Since Zn is considered to be a marker of inflammation^43^, this result also indicates that the calcium phosphate deposition in the medullary interstitial is a pathological process.

### Mean XRF voxel-spectrum

Next to the elemental distribution maps, complete local XRF spectral information is necessary to obtain detailed information on the chemical composition of local features. This might reveal rare characteristics appearing only in particular sample locations/voxels ("needle in the haystack problem”). Since we collect the full XRF spectrum in each measured pixel during data collection, next to the tomograms of the predefined elements, it is also possible to reconstruct the full XRF spectrum in each voxel of the tomogram. In other words, we can include a spectral dimension to the reconstructed dataset, which can be used to extract the mean XRF spectrum of any chosen sample area or volume. Such complete hyperspectral tomography reconstruction was realized for each energy channel of the measured XRF spectra. At 10 keV excitation energy, this results in 1000 sinograms for each virtual slice, and the reconstruction process, even with the MLEM iterative method and multi-core, is becoming quite time-consuming. For example, the reconstruction of the high-resolution 3D hyperspectral XRF tomogram (with the energy as the 3rd dimension) took ~ 1 h by MLEM for the Arabidopsis seed.

**Figure 6:**
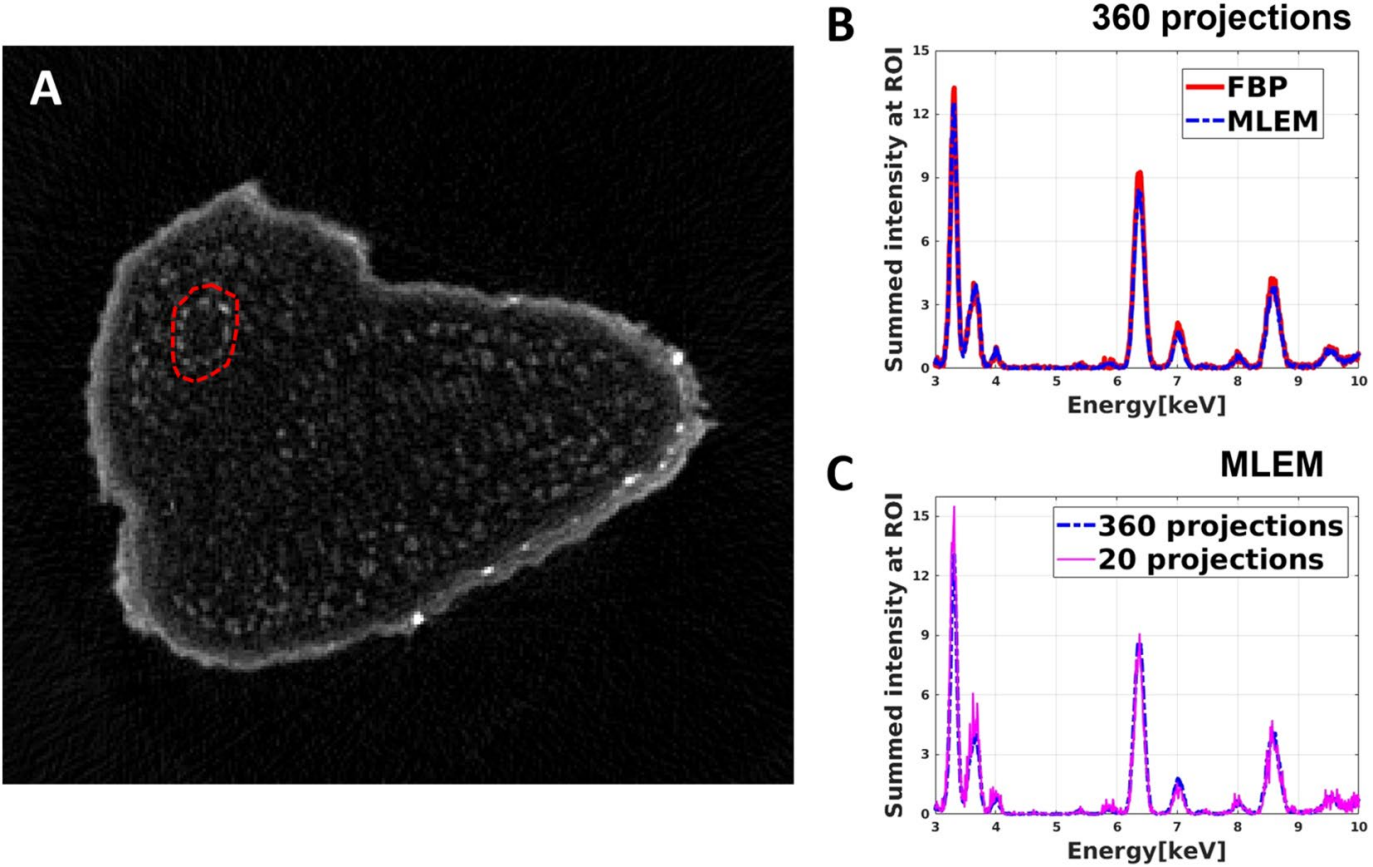
Extraction of the mean XRF spectra of a chosen area in a virtual slice of the Arabidopsis seed. (**A**) Reconstructed high-resolution virtual slice of the sum XRF spectra of the Arabidopsis seed. (**B**) Comparison of the mean XRF spectra of the selected area, marked by a red dashed circle in (**A**), obtained by FBP (red curve) and MLEM (blue curve) algorithm. (**C**) The mean XRF spectra of the selected area obtained by MLEM from 360 projections (blue curve) and from 20 projections (fuchsine curve).

Figure 6A shows the virtual slice of the Arabidopsis seed reconstructed from the sum-XRF spectra of the dataset containing 360 projections. The mean XRF spectra extracted from the area marked by a red circle in Fig. 6A by FBP and MLEM can be seen in Fig. 6B, C. The mean XRF spectra of the chosen region obtained by FBP and MLEM do not show a significant difference; the total intensities of the XRF spectra agree within 5%. As such, in the case of high-resolution tomography, FBP is a good compromise to obtain local spectral information within a limited reconstruction time.

As the next step, we selected equiangularly 20 projections out of the 360 ones to simulate a sparse tomography in identical experimental conditions. This allows comparing the mean XRF spectra obtained by sparse and high-resolution tomographies. In the case of sparse tomography, FBP cannot be used for data reconstruction due to the low number of projections. As such, we reconstructed the sparse tomogram for each energy channel by MLEM. The mean XRF spectrum of the same region as before (red circle in Fig. 6A) was calculated from this sparse tomography dataset. Figure 6C shows that the two mean XRF spectra agree well, which illustrates that the MLEM algorithm provides reliable hyperspectral tomograms and mean XRF spectra even for a severely limited number of projections.

**Figure 7:**
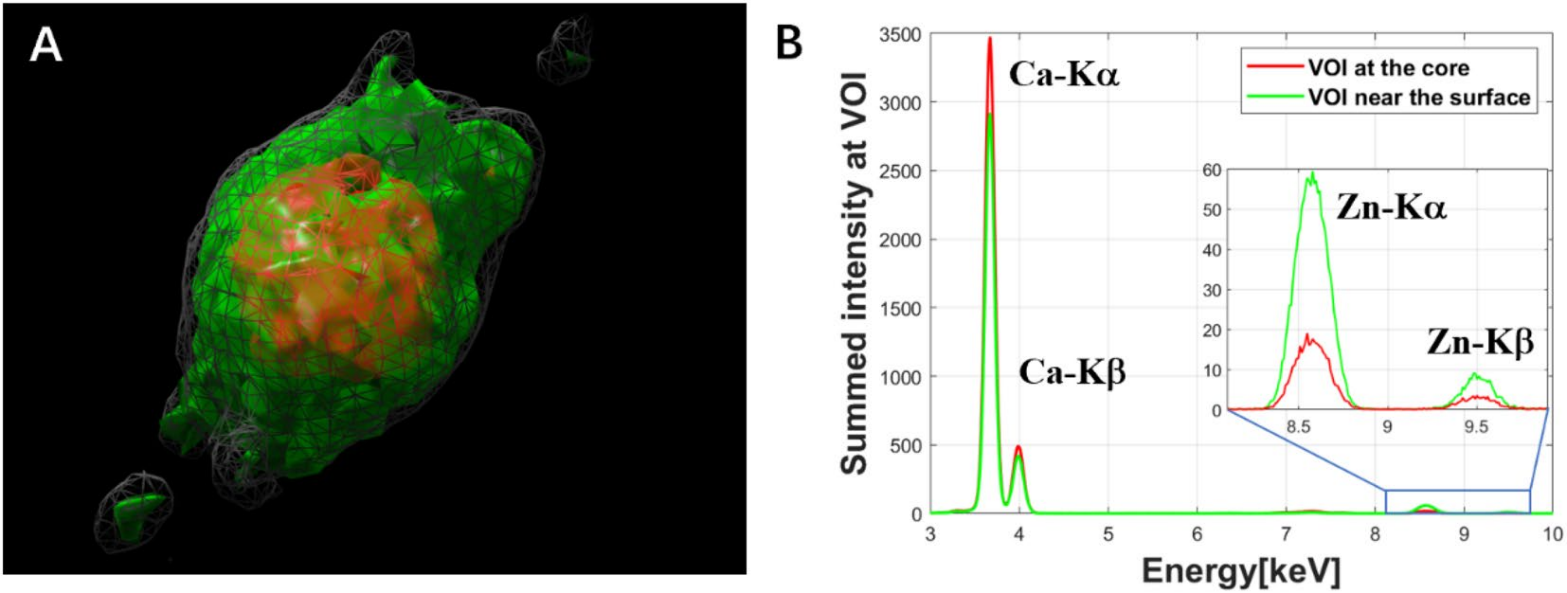
Extraction of the mean XRF spectra within the volume of interest of the reconstructed calcification micro-sphere shown in Fig. 5D,E. (**A**) Reconstructed result by MLEM algorithm from the total intensity of the XRF spectra, grey volume: reconstructed total sphere volume, red and green volumes: core and surface rim regions, respectively. (**B**) Comparison of the mean XRF spectra of the core (red) and surface rim (green) volumes.

The mean XRF spectrum within a selected volume of interest of the calcification sphere (shown in Fig. 5D) of the renal papilla is demonstrated in Fig. 7. The reconstruction process for the 4D hyperspectral tomography dataset (with the energy as the 4th dimension) took around 1 h by the MLEM algorithm. To obtain the mean XRF spectra within a volume of interest, we selected the 3D volumes of interest by ImageJ, a freeware frequently used by our users. We applied this mask to each energy channel in the reconstructed 4D dataset. The extracted mean XRF spectra shown in Fig. 7B reveal that Ca is the dominant element in the core of the calcification micro-sphere (red XRF spectrum), which also contains Zn. Within the surface rim (indicated by green in Fig. 7A and green XRF spectrum in Fig. 7B), the increased Zn to Ca ratio, due to the threefold larger Zn and ~ 20% smaller Ca content, provides evidence of the association of Zn to the pathological process.

## Discussion

The proposed holistic multi-scale and multimodal scanning X-ray tomography workflow, implemented at Nanoscopium, was tested on mesoscale samples. To tackle the scientific problems presented in the recent paper, multi-scale XRF- and scanning absorption contrast tomography were the best-adapted imaging modalities.

The best compromise for semi-automatic on-site tomography reconstruction for sparse-, local- and high-resolution scanning X-ray tomography is proved to be the MLEM algorithm if it is benefited from the apt early stopping strategy. This algorithm also efficiently handles the missing wedge sampling conditions. The FBP algorithm is, in general, not adapted for the reconstruction of sparse and missing wedge tomographies since the measured dataset does not fulfill the optimal Nyquist sampling criterion. In the case of high-resolution hyperspectral tomography, which results in large 3D/4D datasets, the figure of merit is a compromise between the reconstructed data quality and the reconstruction time. Here, FBP is the best compromise to obtain, within a limited reconstruction time, reliable mean XRF-spectra from regions/volumes chosen from the reconstructed XRF hyper-spectral tomograms. The semi-automatic use of these two algorithms according to the above-detailed conditions permits obtaining a flexible semi-automatic workflow providing good quality on-site reconstruction for various samples in diverse experimental conditions. Due to its flexibility, our approach is especially well suited for on-the-fly tuning of the experiments to study features of interest progressively at different length scales.

We must note that scanning XRF imaging is generally a semi-quantitative approach. However, in the present paper, we presented the 2D and 3D elemental intensity distributions (number of characteristic X-ray Fluorescence photons per given dwell time). As the next step, we intend to fully integrate the quantification method in the semi-automatic user-friendly data-processing pipeline and to provide it for all scanning XRF tomography experiments as an option. For this, the measurement conditions will be calibrated by an adequate reference sample, and simultaneous scanning X-ray absorption tomography will be used for self-absorption correction in each voxel of the tomogram for each reconstructed element.

The robust scanning tomography method implemented at the Nanoscopium beamline opens the way for non-expert users towards routine non-destructive multi-length-scale characterization of complex samples during a standard beam-time of 3–5 days. The reconstructed tomograms can be treated by widely used freewares such as ImageJ, Chimera, etc. This allows straightforward data handling for users during and after their experiments. The workflow provides the possibility to study relevant 3D micro-features of several mesoscale samples during a routine user experiment, even at 3rd generation synchrotrons. Next to XRF and scanning absorption tomography, we have also extensively tested the workflow for scanning phase contrast- and X-ray diffraction tomography studying perovskite samples and bio-mineralization. These results will be presented in a separate paper. Moreover, the application of our approach for XANES tomography, which can be considered as a variant of hyperspectral tomography^44^, is straightforward.

Such a robust semi-automatic flexible scanning multimodal tomography workflow will be a scientific game-changer at emerging 4th generation synchrotron sources, where data throughput of scanning hard X-ray techniques is boosted by ~ two orders of magnitude. The new possibility to explore a multitude of sample characteristics simultaneously, with high analytical sensitivity, at hierarchical length-scales in 3D, in a statistically significant manner in meso- and micro-scale samples, will revolutionize a wide range of scientific fields in ways that we can currently only dream of and will provide a unique complement to already existing state-of-the-art multi-scale and fast full-field X-ray tomography techniques^45–47^. These high through-put scanning X-ray imaging techniques will also complement emerging state-of-the-art laboratory scanning charged-particle microscopy and tomography and other conventional laboratory microscopy techniques providing 2D/3D elemental and morphology information with nanometer resolution. For example, scanning electron microscopy^48^ has limited analytical sensitivity (~ 0.1–1%) for the analysis of heavy elements, and its small depth of information (a few microns) limits its non-invasive (without sample sectioning) application to surface studies of large samples or the 3D study of light, major, and minor components of tiny, micrometer-sized samples. Due to sample radiation damage caused by charged-particle microscopies, multiple measurement on the very same sample region is often problematic. Scanning hard XRF imaging and tomography has high analytical sensitivity (with trace, ≤ ppm detection limit) for transition metals and heavier elements. Moreover, their large information depth of several tens/hundreds of microns permits the non-invasive multilength-scale 3D study of mesoscale samples. Multiple measurements and hence, multi-scale and local tomography is readily available by scanning X-ray imaging techniques, where radiation damage is smaller than by charged particle microscopies.

## Methods

### Data acquisition

The experiments were performed at the KB-based nanoprobe station of the Nanoscopium beamline of Synchrotron Soleil^21^. Two energy-dispersive silicon drift detectors, placed at 120° to the incident beam path, collected the X-ray fluorescence (XRF) spectra. The intensity of the incident and the transmitted beam has been collected in each pixel by two Si diodes placed before and behind the sample, respectively. An RT100 air-bearing rotation stage has been used for tomography measurements. The experiments have been performed by the Flyscan architecture^1^.

In the case of scanning X-ray single slice tomography, the number of angular projections has been chosen according to the Nyquist sampling condition (see Eq. 3) in order to preserve the spatial resolution along the horizontal direction in the reconstructed tomograms.

The medium resolution 3D elemental distribution of the Arabidopsis seed has been obtained by sparse XRF tomography at 11 keV excitation X-ray energy. We collected 20 projections at 18 degrees angular intervals over 360°. These scanning parameters are proposed by default for sparse tomography by our workflow since, according to our experience with multiple samples and imaging modalities, they are proved to be the best-balanced parameters for almost all experimental conditions. At each projection angle, a total area of 581 × 445 μm^2^ has been scanned with 2 μm pixel size and 20 ms dwell time in continuous scanning mode. The collection of the full XRF sparse tomography dataset of this mesoscale sample took 8 h 40 min. For high-resolution single slice tomography, we measured 360 angular projections over 360° with a lateral step size of 2 μm and a dwell time of 20 ms/pixel, with a total measurement time of 35 min. For high-resolution 2D projection imaging, a field of view of 375 × 434 μm^2^ was scanned with a step of 500 nm and a dwell time of 40 ms per pixel. The total acquisition time was 7 h 30 min.

In the case of the mouse renal papilla sample, the sparse tomography was performed at 12 keV. A 586 × 272 μm^2^ region of the sample was mapped at 22 angular positions over 360° with a lateral step size of 2 μm and an exposure time of 20 ms/pixel, with a total measurement time of 5 h. This is followed by a high-resolution local tomography of a local sample volume with 65 angular positions over 360° with a dwell time of 40 ms/pixel and a uniform lateral step size of 500 nm, covering a total area of 67 × 16 μm^2^. The total acquisition time was 3 h.

### Data processing

The elements present in the investigated samples were identified from the sum-spectra of all angular projections. The sum XRF spectra were fitted by the PyMCA software^49^ for the identification of the elements present in the sample. The elemental distribution maps and sinograms were then extracted from the raw dataset by integrating pre-selected spectral regions of interest corresponding to the detected elements. The transmitted and incident beam intensity ratio at each pixel gives access to the sample absorption maps and sinograms. The sinograms were then reconstructed either by the MLEM or FBP algorithms. The overall reduction process is performed automatically with a robust in-house MATLAB code. The reconstructed volumetric data were exported from MATLAB as 16-bit z-stacks and imported either to ImageJ^50^ for analysis or to Chimera^51^ for 3D visualization.

The overall data processing is performed on a workstation with an Intel® Xeon® Processor E5-2630 v3 @ 2.40 GHz × 32 CPU with 125.8 GB of system RAM.

### Reconstruction algorithms

Two different tomography reconstruction algorithms have been integrated into the workflow: the FBP^42,52,53^ and MLEM^54^ methods. The first, which is an analytical filtering inversion technique, is the most commonly used method in routine tomography reconstruction. It is the fastest method, exploiting fast Fourier transforms. However, FBP suffers from a lack of robustness when the measurements are sparse, of low contrast, or noisy^55,56^, which is often the case in XRF tomography. MLEM is an iterative algorithm that explicitly accounts for the noise affecting the data and imposes positivity on the estimated pixels. MLEM was initially developed for the analysis of positron emission tomography (PET) data. It assumes a Poisson distribution of the acquired photon statistics and thus might be more noise-tolerant than FBP^22^. MLEM belongs to the class of majorization-minimization algorithms^57^. By construction, it decreases the negative log-likelihood of the estimated image of the given data monotonically. Moreover, it involves only simple multiplicative updates in such a way that the estimated image has positive pixel values, if all the pixels of the starting assumption image are positive. The MLEM algorithm provides good performance with sparse datasets, and the streak artifacts can be reduced as long as it is combined with an appropriate early stopping strategy, as is described hereafter.

### Determination of the number of iterations: automatic early stopping strategy for MLEM

Although the loss of the MLEM algorithm decreases along with iterations, there is also an increase in the noise amplitude, which significantly impacts the final reconstruction results. This is typical for non-regularized reconstruction methods. Therefore a so-called early stopping strategy with a well-tuned criterion is crucial and must be integrated into the workflow^58^. We implemented the figure of merit about the Normalized Root Mean Square Error Deviation (NRMSED) as an indicator:

1 $$\begin{array}{*{20}c} {NRMSED = \sqrt {\frac{{\mathop \sum \nolimits_{i = 1}^{N} \left( {x\left( i \right) - \tilde{x}\left( i \right)} \right)^{2} }}{{\mathop \sum \nolimits_{i = 1}^{N} x\left( i \right)^{2} }}} } \\ \end{array}$$

where *x*(*i*) is the sinogram of the measured data, $\tilde{x}\left( i \right)$ is the estimated sinogram by MLEM and N is the pixel number in the sinogram. NRMSED indicates the accordance between the reconstructed and measured data. In addition to this, information about the derivative of NRMSED between two successive iterations should also be considered, and the ratio R is therefore defined as:

2 $$\begin{array}{*{20}c} {R = \frac{\Delta NRMSED}{{NRMSED}}} \\ \end{array}$$

where $\Delta NRMSED = NRMSED^{\left( i \right)} - NRMSED^{{\left( {i - 1} \right)}}$ is the derivative of NRMSED. This similarity increases monotonically with the number of iterations. A threshold is set to terminate the algorithm when the evolution curve of R is close to zero: $R \ge threshold$. We have chosen − 0.15% as *threshold*, since this value, obtained as average over numerous test data sets, gave robust and rapid results for all tested data sets and imaging modalities.

In the implementation of our workflow, a relatively large number (~ e.g., 200) of iterations is preset to limit the maximum reconstruction time. The reconstruction of the tomograms using the MLEM algorithm is performed slice by slice, and the ratio R is calculated for each slice after each iteration. The algorithm terminates automatically for a given virtual slice after reaching the threshold and proceeds to reconstruct the next slice. Optionally, the reconstruction could perform a few additional iterations after reaching the R threshold. According to our experience, the algorithm generally reaches the $R \ge threshold$ condition around 20 iterations, well before 200 iteration steps.

The process of the optimization of the iteration number can be summarized as follows:

1. For all the slices, set the *threshold* to − 0.15% and the initial number of iterations to a relatively large value (~ 200);
2. Start the reconstruction for the first slice using the MLEM algorithm while calculating the ratio R for each iteration;
3. Stop the iteration when the condition $R \ge threshold$ is reached;
4. Repeat (2)–(3) for all slices.

This robust automatic reconstruction process provides exploitable results even for 5 angular projections, and it assures that even inexperienced users in synchrotron-based imaging/tomography can reconstruct their tomographic dataset on-site.

### Spatial resolution evaluation

#### Nyquist angular sampling condition

During the experiment, a reasonable trade-off must be reached between the acquisition time and spatial resolution. For the analytical reconstruction methods, the number of projections has to satisfy the Nyquist angular sampling condition^59^ in order to preserve the spatial resolution along the horizontal direction in the reconstructed tomogram:

3 $$\begin{array}{*{20}c} {n_{proj} = \frac{\pi }{2}N} \\ \end{array}$$

where $n_{proj}$ is the number of projections over 180°, and N is the number of pixels along the scanning direction.

The corresponding maximum resolvable spatial frequency $f_{res}$ in the Fourier domain can be written as:

4 $$\begin{array}{*{20}c} {f_{res} = \frac{1}{\pi }\frac{{n_{proj} }}{PN}} \\ \end{array}$$

where P is the pixel size.

The resolution limit $R$ in direct space is then:

5 $$\begin{array}{*{20}c} {R = \frac{\pi }{2}\frac{PN}{{n_{proj} }}} \\ \end{array}$$

This resolution limit decreases with increasing number of projections.

#### Fourier ring correlation for spatial resolution estimation

The most common method to estimate spatial resolution is the knife-edge technique. However, in the case of sparse tomography of highly heterogeneous samples, it can be challenging to find a sharp edge for the knife-edge method. To overcome this difficulty, the Fourier ring correlation (FRC)^60–62^ method can be used as a general approach.

For the iterative reconstruction methods, the estimation of the achievable spatial resolution in the function of the number of projections can be described by the Fourier ring correlation approach. FRC estimates this by measuring the normalized cross-correlation of two independent datasets of the sample. The spatial frequency elements at different radii can be integrated circularly in the frequency domain:

6 $$\begin{array}{*{20}c} {FRC_{12} \left( r \right) = \frac{{\mathop \sum \nolimits_{{r_{i} \in r}} F_{1} \left( {r_{i} } \right) \cdot F_{2} \left( {r_{i} } \right)^{*} }}{{\sqrt {\mathop \sum \nolimits_{{r_{i} \in r}} |F_{1} \left( {r_{i} } \right)|^{2} \cdot \mathop \sum \nolimits_{{r_{i} \in r}} |F_{2} \left( {r_{i} } \right)|^{2} } }}} \\ \end{array}$$

where $r_{i}$ is the *i*th frequency element at radius $r$, $F_{1}$ and $F_{2}$ denote the Fourier transform of the two reconstructed tomograms.

At a specified cut-off threshold, the FRC curve drops below the threshold, indicating an indistinguishable signal-to-noise ratio. This defines the spatial resolution.

Theoretically, the two data sets used in FRC calculation should be formed by independent measurement^63^, but this can be impractical, especially in the high-resolution scanning experiments, where the acquisition time is generally several hours at 3rd generation synchrotrons. To overcome this problem, the two subsets used for FRC calculation were extracted from the high-resolution tomography sinogram, from which independent tomographic images of the same slice were reconstructed. This resolution estimation method is mostly applied to 2D high-resolution datasets.

## Ethics approval

*Arabidopsis thaliana* seeds: The research on the wild-type Col0 seed *Arabidopsis Thaliana* seed complies with relevant institutional, national, and international guidelines and legislation. The wild-type *Arabidopsis thaliana* seeds had been provided by C. Curie, and is the same as described by Carrió-Seguí, À., Romero, P., Curie, C. et al. “Copper transporter COPT5 participates in the crosstalk between vacuolar copper and iron pools mobilization.” In *Sci Rep 9, 4648 (2019)* https://rdcu.be/cROAu*.* Renal papilla samples: All studies were performed in accordance with the European Union, NIH guidelines (Comité d'Ethique en Experimentation Charles Darwin C2EA-05) and all of our methods are reported as recommended by ARRIVE guidelines as previously described. The project was authorized by the Health Ministry and local Ethics Committee (authorization 11420 2017092015335292).

## Supplementary Information

Supplementary Information.

## Data Availability

All data needed to evaluate the conclusions in the paper are present in the paper and/or the Supplementary Materials. Additional data related to this paper will be available from the authors upon reasonable request and in accordance with the Journal policy described in the Instructions for Authors (Availability of materials and data).
