# Supplementary material for: Towards routine 3D characterization of intact mesoscale samples by multi-scale and multimodal scanning X-ray tomography
Source: Sci Rep. 2022 Oct 8;12:16924. doi: 10.1038/s41598-022-21368-0 (PMC9547857; doi:10.1038/s41598-022-21368-0)
Supplement: Supplementary file 1 — Supplementary Information. [file 41598_2022_21368_MOESM1_ESM.docx]

Supplementary Materials for

Towards Routine 3D Characterization of Intact Mesoscale Samples by Multi-scale and Multimodal Scanning X-ray Tomography

Authors

Ruiqiao Guo, Andrea Somogyi, Dominique Bazin, Elise Bouderlique, Emmanuel Letavernier, Catherine Curie, Marie-Pierre Isaure, Kadda Medjoubi

This file includes:

Fig. S1


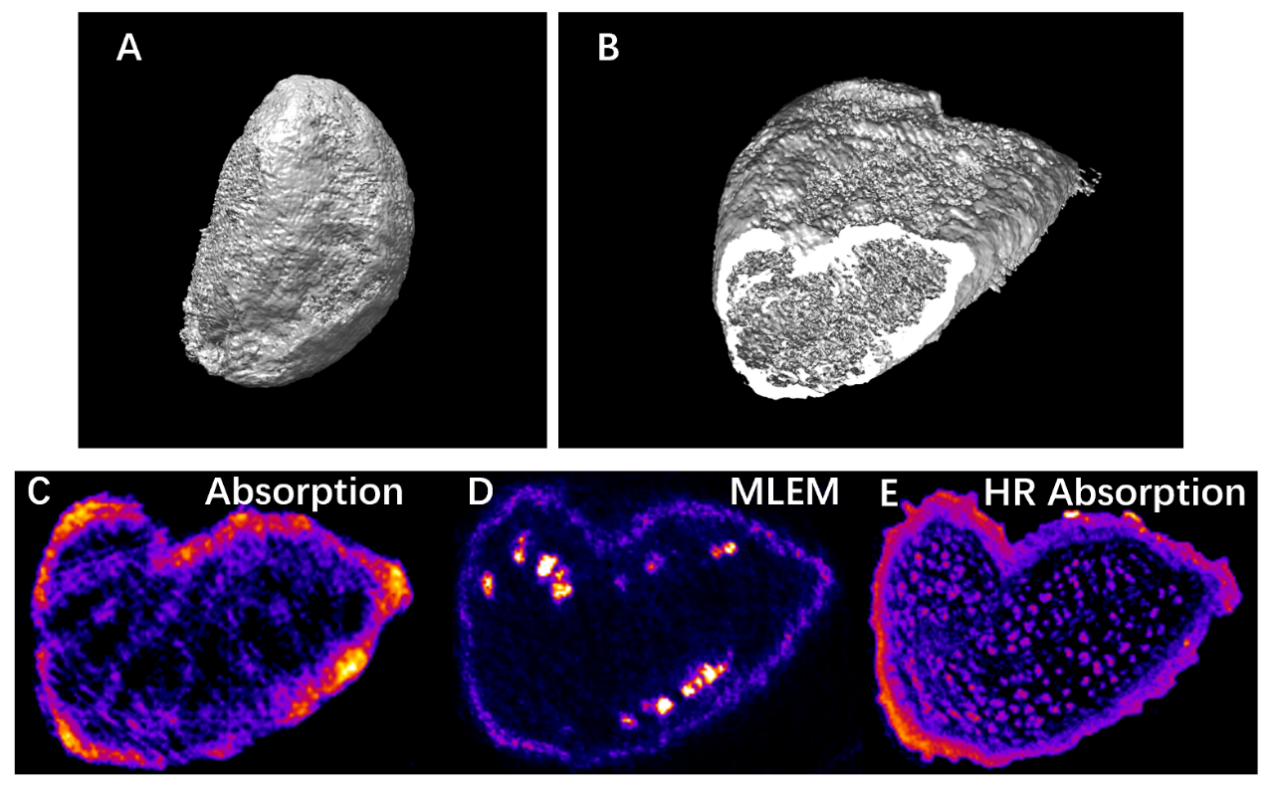


**Fig. S1. Reconstruction of the scanning X-ray absorption tomography of the Arabidopsis thaliana seed, collected simultaneously with XRF tomography.**

**(A)** 3D volume rendering of X-ray absorption reconstructed from the 20-projection dataset with MLEM algorithm. Cut-off view **(B)** and the reconstructed virtual slice **(C)** of the absorption tomogram at the altitude marked by the red line in Fig. 2. **(D)** Mn distribution within the same virtual slice as B and C obtained with MLEM algorithm from the XRF tomography dataset (already shown in Fig. 3D). **(E)** Reconstruction result of the high-resolution X-ray absorption sinogram.
